# Supplementary material for: Modelling Skylarks (Alauda arvensis) to Predict Impacts of Changes in Land Management and Policy: Development and Testing of an Agent-Based Model
Source: PLoS One. 2013 Jun 6;8(6):e65803. doi: 10.1371/journal.pone.0065803 (PMC3675089; doi:10.1371/journal.pone.0065803)
Supplement: Supporting Information S4 — The skylark ODdox as a zipped archive. (ZIP) [file pone.0065803.s004.zip › Skylark_ODdox/class_t_skylark_list.html]

ALMaSS Skylark ODdox: TSkylarkList< T > Class Template Reference


ALMaSS Skylark ODdox
 2.0


- Main Page
- Related Pages
- Classes
- Files

- Class List
- Class Index
- Class Hierarchy
- Class Members

Public Member Functions

TSkylarkList< T > Class Template Reference

`#include <skylarks_all.h>`

List of all members.

|  |  |
| --- | --- |
| Public Member Functions | |
| double | Probe (probe\_data \*p\_TheProbe) |
|  | TSkylarkList () |

---

## Constructor & Destructor Documentation

template<class T >

|  |  |  |  |  |
| --- | --- | --- | --- | --- |
| TSkylarkList< T >::TSkylarkList | ( |  | ) |  |

inline

: vector < T > ()

{

}

---

## Member Function Documentation

template<class T >

|  |  |  |  |  |  |
| --- | --- | --- | --- | --- | --- |
| double TSkylarkList< T >::Probe | ( | probe\_data \* | *p\_TheProbe* | ) |  |

inline

References AnimalPosition::m\_EleType, probe\_data::m\_NoAreas, probe\_data::m\_NoEleTypes, probe\_data::m\_NoFarms, probe\_data::m\_NoVegTypes, probe\_data::m\_Rect, probe\_data::m\_RefEle, probe\_data::m\_RefFarms, probe\_data::m\_RefVeg, AnimalPosition::m\_VegType, AnimalPosition::m\_x, rectangle::m\_x1, rectangle::m\_x2, AnimalPosition::m\_y, rectangle::m\_y1, and rectangle::m\_y2.

{

// Counts through the list and goes through each area to see if the animal

// is standing there and if the farm, veg or element conditions are met

AnimalPosition Sp;

double NumberSk = 0;

// Four possibilites

// either NoVegTypes or NoElementTypes or NoFarmTypes is >0 or all==0

if ( p\_TheProbe->m\_NoFarms != 0 )

{

for ( unsigned j = 0; j < this->GetItemsInContainer(); j++ )

{

Sp = this->Get( j )->SupplyPosition();

unsigned Farm = this->Get( j )->SupplyFarmOwnerRef();

for ( unsigned i = 0; i < p\_TheProbe->m\_NoAreas; i++ )

{

if ( ( Sp.m\_x >= p\_TheProbe->m\_Rect[i].m\_x1 ) && ( Sp.m\_y >= p\_TheProbe->m\_Rect[i].m\_y1 )

&& ( Sp.m\_x < p\_TheProbe->m\_Rect[i].m\_x2 ) && ( Sp.m\_y < p\_TheProbe->m\_Rect[i].m\_y2 ) )

for ( unsigned k = 0; k < p\_TheProbe->m\_NoFarms; k++ )

{

if ( p\_TheProbe->m\_RefFarms[k] == Farm )

NumberSk++; // it is in the square so increment number

}

}

}

}

if ( p\_TheProbe->m\_NoEleTypes != 0 )

{

for ( unsigned j = 0; j < this->GetItemsInContainer(); j++ )

{

Sp = this->Get( j )->SupplyPosition();

for ( unsigned i = 0; i < p\_TheProbe->m\_NoAreas; i++ )

{

if ( ( Sp.m\_x >= p\_TheProbe->m\_Rect[i].m\_x1 ) && ( Sp.m\_y >= p\_TheProbe->m\_Rect[i].m\_y1 )

&& ( Sp.m\_x < p\_TheProbe->m\_Rect[i].m\_x2 ) && ( Sp.m\_y < p\_TheProbe->m\_Rect[i].m\_y2 ) )

for ( unsigned k = 0; k < p\_TheProbe->m\_NoEleTypes; k++ )

{

if ( p\_TheProbe->m\_RefEle[k] == Sp.m\_EleType )

NumberSk++; // it is in the square so increment number

}

}

}

}

else

{

if ( p\_TheProbe->m\_NoVegTypes != 0 )

{

for ( unsigned j = 0; j < this->GetItemsInContainer(); j++ )

{

Sp = this->Get( j )->SupplyPosition();

for ( unsigned i = 0; i < p\_TheProbe->m\_NoAreas; i++ )

{

if ( ( Sp.m\_x >= p\_TheProbe->m\_Rect[i].m\_x1 ) && ( Sp.m\_y >= p\_TheProbe->m\_Rect[i].m\_y1 )

&& ( Sp.m\_x < p\_TheProbe->m\_Rect[i].m\_x2 ) && ( Sp.m\_y < p\_TheProbe->m\_Rect[i].m\_y2 ) )

{

for ( unsigned k = 0; k < p\_TheProbe->m\_NoVegTypes; k++ )

{

if ( p\_TheProbe->m\_RefVeg[k] == Sp.m\_VegType )

NumberSk++; // it is in the square so increment number

}

}

}

}

}

else // both must be zero

{

for ( unsigned j = 0; j < this->GetItemsInContainer(); j++ )

{

Sp = this->Get( j )->SupplyPosition();

for ( unsigned i = 0; i < p\_TheProbe->m\_NoAreas; i++ )

{

if ( ( Sp.m\_x >= p\_TheProbe->m\_Rect[i].m\_x1 ) && ( Sp.m\_y >= p\_TheProbe->m\_Rect[i].m\_y1 )

&& ( Sp.m\_x < p\_TheProbe->m\_Rect[i].m\_x2 ) && ( Sp.m\_y < p\_TheProbe->m\_Rect[i].m\_y2 ) )

NumberSk++; // it is in the square so increment number

}

}

}

}

return NumberSk;

}

---

The documentation for this class was generated from the following file:

- skylarks\_all.h


- TSkylarkList
- Generated on Thu Jan 10 2013 13:15:38 for ALMaSS Skylark ODdox by
   1.8.1.1
